# Structural Insights into the Nonmutagenicity of 2-Haloacetophenone

**DOI:** 10.3390/molecules30061264

**Published:** 2025-03-12

**Authors:** Hunmin Jung, Naveen Kumar Rayala, Ritesh Pal, Seongmin Lee

**Affiliations:** 1 The Department of Pharmaceutical Sciences, School of Pharmacy, University of Connecticut, Storrs, CT 06269, USA; 2 The Division of Chemical Biology and Medicinal Chemistry, College of Pharmacy, The University of Texas at Austin, Austin, TX 78712, USAritesh.pal@austin.utexas.edu (R.P.)

**Keywords:** DNA damage, alkylation, translesion synthesis, mutagenesis

## Abstract

A wide variety of endogenous and exogenous alkylating agents covalently modify DNA to produce N7-alkyl-2′-deoxyguanosine (N7-alkylG) adducts as major DNA lesions. The mutagenic potentials of many N7-alkylG adducts with an intercalatable moiety remain poorly understood. We have discovered that the antiriot agent 2-chloroacetophenone readily reacts with dG to produce N7-acetophenone-dG adducts, implicating the genotoxic properties of 2-chloroacetophenone. 2-Chloroacetophenone, however, has been found to be nonmutagenic in both bacterial and mammalian cells. To gain insights into the nonmutagenic nature of N7-acetophenone-dG, we prepared N7-acetophenone-dG-containing oligonucleotide via 2′-fluorine-mediated transition-state destabilization and conducted kinetic and structural studies of human DNA polymerase eta (polη) incorporating nucleotide opposite 2′-F-N7-acetophenone-dG. The kinetic experiments reveal that the presence of the lesion at the templating position greatly hinders nucleotide incorporation. A crystal structure of polη bound to a nonhydrolyzable dCTP analog opposite 2′-F-N7-acetophenone-dG shows that the templating N7-acetophenone-dG is in a *syn* conformation, precluding binding of an incoming nucleotide in the catalytic site. These unusual conformations explain the observed inefficient incorporation of nucleotide opposite the lesion. Our studies suggest that certain bulky N7-alkylG lesions adopt a *syn* conformer and present an intercalatable moiety into the nascent base-pairing site, deterring nucleotide incorporation and thus lowering mutagenicity.

## 1. Introduction

Nucleobases in DNA are susceptible to nonenzymatic modification by a range of endogenous and exogenous alkylating agents. N7 of guanine is the most nucleophilic atom in DNA and preferentially reacts with varying alkylating agents including *S*-adenosylmethionine (SAM), nitrogen mustards, styrene oxide, nicotine nitrosamine ketone, and aflatoxin B1 (AFB1) [1]. While small N7-alkylG lesions such as N7-methylguanine is little to nonmutagenic [2], certain bulky N7-alkylG lesions such as N7-AFB1-G are highly mutagenic and carcinogenic [3]. For example, N7-AFB1-G adducts have shown to preferentially induce G-to-T mutations and contribute to the etiology of liver cancers [4,5].

**Figure 1:**
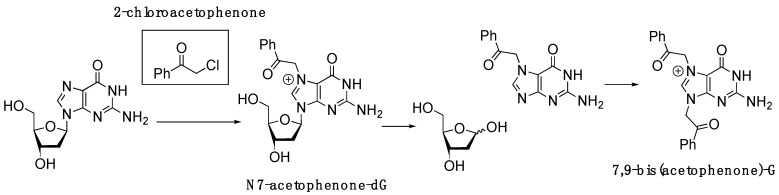
Reaction of 2-chloroacetophenone with dG. The N7-acetophenone-dG adduct would undergo spontaneous depurination to generate abasic site and N7-acetophenone-G nucleobase, which can react with another 2-chloroacetophenone to produce 7,9-bis(acetophenone)-G.

2-Chloroacetophenone, also known as phenacyl chloride, is utilized as a riot-control or tear agent [6]. Inhalation exposure to this chemical induces burning of the eyes, accompanied by lacrimation [7]. It is recognized as a potent irritant to both the eyes and skin. Studies have indicated that 2-chloroacetophenone does not exhibit carcinogenic activity in male rats or male and female mice [8]. In the course of guanine alkylation investigations, we have observed that dG readily reacts with 2-chloroacetophenone at ambient temperatures to produce 7,9-bis(acetophenone)-dG adducts, presumably via an intermediacy of N7-acetophenone-dG adducts (Figure 1). Attempts to isolate N7-acetophenone-dG failed due to the chemical instability of the adduct during silica gel column chromatography. N7,9-bis(acetophenone)guanine was predominantly produced instead, indicating the N7-dG adduct formation followed by depurination. The notable reactivity of 2-chloroacetophenone towards dG prompted our inquiry into the mechanism underlying the non-mutagenic properties of the lesion produced by this reactive alkylating agent.

**Figure 2:**
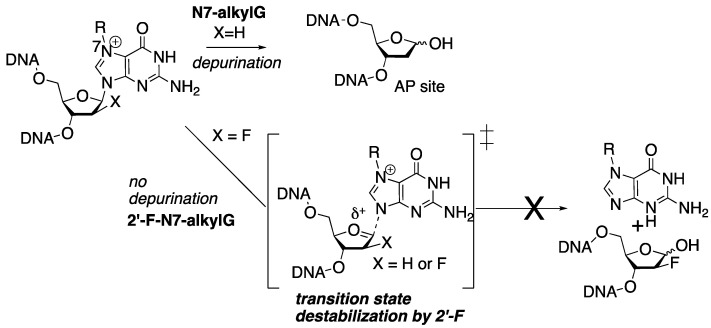
Stabilization of N7-alkyl-dG adducts by 2′-F. The spontaneous depurination of N7-alkyl-dG lesions is prevented by the destabilization of an oxocarbenium ion by fluorine.

Studying the biochemical and mutagenic properties of N7-alkylG lesions presents challenges due to their susceptibility to spontaneous depurination, leading to the formation of abasic sites (Figure 2). While N7-alkylG lesions in DNA have half-lives of several hours to days, N7-alkylG-containing nucleosides rapidly undergo depurination [9]. To address this chemical stability issue, we previously devised a strategy involving 2′-fluorine-mediated transition-state destabilization [2,10]. The incorporation of 2′-F near the glycosidic bond serves to destabilize the oxocarbenium ion-like transition state, thus impeding glycosidic bond cleavage (Figure 2) [11]. Leveraging the 2′-F technology, we have conducted kinetic and structural investigations of DNA polymerase catalyzing across 2′-F-N7-alkylG adducts.

Human DNA polymerase eta (polη) is a member of the Y-family DNA polymerases and has been implicated in the bypass of various types of DNA damage, including cyclobutane pyrimidine dimers, cisplatin-GpG adducts, 8-oxoguanine, and O6-methylguanine [12,13]. Mutations in the POLH gene can lead to xeroderma pigmentosum type V (XP-V) [14], a condition characterized by hypersensitivity to UV radiation and an increased mutation rate. Similar to other Y-family DNA polymerases, polη does not undergo an open-to-closed conformational change during translesion synthesis [15]. With a large solvent access area and a relatively rigid active site conformation, polη can accommodate small to medium-sized DNA lesions. We recently reported that polη bypasses some N7alkylG lesions including N7-methylG (N7mG) [16], N7-benzylG (N7BnG) [17], and N7-nitrogen half-mustardG (NHMG) [18].

Herein, we report the synthesis of 2′-F-N7-acetophenone-dG phosphoramidite and its incorporation into oligonucleotide via solid-phase DNA synthesis. We also describe kinetic studies of polη catalyzing across 2′-F-N7-acetophenone-G lesion. Furthermore, we present a crystal structure of polη in the presence of templating N7-acetophenone-dG (N7AcPhG) and incoming nonhydrolyzable dCTP analog. The first structure of translesion synthesis (TLS) DNA polymerase bypassing N7AcPhG provides new insights into the nonmutagenicity of 2-chloroacetophenone.

## 2. Results and Discussion

### 2.1. Synthesis of 2′-Deoxy-2′-F-N7-Acetophenone-dG-Containing Oligonucleotide

**Figure 3:**
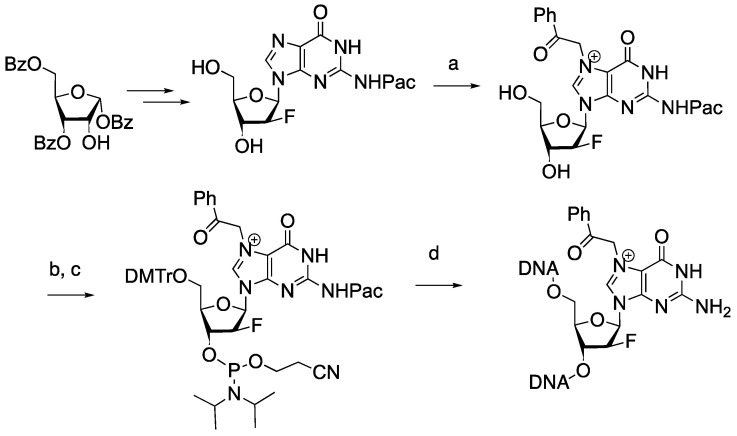
Preparation of 2′-F-N7-acetophenone-dG-containing DNA. Reagents and conditions: (a) 2-bromoacetophenone, DMF, 25 °C, 18 h; (b) DMTrCl, pyridine, 25 °C, 2 h; (c) 4,5-Dicyanoimidazole, (*i*-Pr_2_N)_2_P(OC_2_H_4_CN), CH_2_Cl_2_, 25 °C, 1 h; (d) solid-phase DNA synthesis and ultra-mild deprotection conditions (K_2_CO_3_, MeOH, 25 °C).

2′-F-N2-Pac-dG nucleoside was prepared as described previously [10]. The reaction of 2′-F-N2-Pac-dG with 2-bromoacetophenone in DMF at an ambient temperature resulted in the formation of 2′-F-N2-Pac-N7-acetophenone-dG, which was then subjected to sequential tritylation and phsophatidylation (Figure 3). These reactions provided 2′-F-N7-acetophenone-dG phosphoramidite, which was incorporated into oligonucleotide via a solid-phase DNA synthesis. Ultra-mild deprotection conditions (50 mM K_2_CO_3_ in MeOH) were used to remove protective groups in the oligonucleotide to afford 2′-F-N7-acetophenone-dG-containing oligonucleotide.

### 2.2. Polη Bypasses N7AcPhG Lesion with Low Efficiency

**Figure 4:**
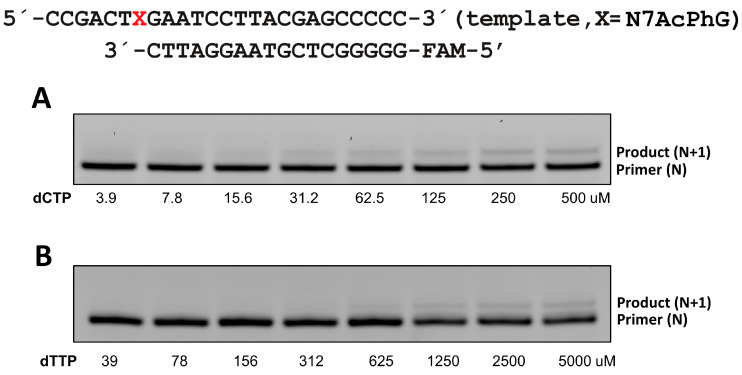
Nucleotide incorporation opposite templating N7AcPhG by polη. Urea PAGE gels showing incorporation of dCTP (**A**) and dTTP (**B**) opposite the lesion. DNA substrate containing N7AcPhG lesion was annealed with a 5′-FAM-labeled primer. Then, the annealed DNA containing templating N7AcPhG was mixed with polη and the reactions were initiated by adding varying concentrations of dCTP or dTTP. Each reaction was conducted for 2–4 min at 23 °C and the quenched samples were separated on 18% denaturing urea PAGE gels. X denotes N7AcPhG.

To assess the catalytic activity of polη replication across N7AcPhG, we determined kinetic parameters (*k_cat_* and *K_m_*) for nucleotide incorporation opposite templating N7AcPhG (Table 1). The insertion of nucleotide opposite N7AcPhG was greatly deterred (Figure 4). The presence of N7AcPhG at the templating position reduced the insertion efficiency for dCTP by ~670-fold compared to templating guanine (45.6 × 10^−3^ s^−1^μM^−1^ vs. 0.068 × 10^−3^ s^−1^μM^−1^ of *k_cat_*/*K_m_*), displaying the impact of bulky N7-alkylG on nucleotide insertion. The templating N7AcPhG also reduced the insertion efficiency for dTTP, but to a lower degree (72-fold, 0.47 × 10^−3^ s^−1^μM^−1^ vs. 0.0065 × 10^−3^ s^−1^μM^−1^ of *k_cat_*/*K_m_*). The decreased insertion efficiency for N7AcPhG:dCTP (correct insertion) was caused by the reduction in both *k_cat_* and *K_m_*, and the decreased insertion efficiency for N7AcPhG:dTTP (incorrect insertion) mainly resulted from the reduced *k_cat_* (74.8 × 10^−3^ s^−1^ vs. 3.6 × 10^−3^ s^−1^). The replication fidelity, the ratio between correct and incorrect insertion, for the catalysis across N7AcPhG was ~9 (Table 1), while that across guanine was ~100, revealing the promutagenic nature of N7AcPhG. The bypass of bulky N7alkylG (N7BnG, N7AcPhG, and NHMG) by polη was error-prone (about 10:1 ratio of *k_cat_*/*K_m_* between correct and incorrect insertions), which is about a 10-fold increase from the undamaged dG whose mutagenic bypass ratio of *k_cat_*/*K_m_* was about 100:1 (45.6 × 10^−3^ s^−1^μM^−1^ vs. 0.47 × 10^−3^ s^−1^μM^−1^).

### 2.3. N7AcPhG at the Templating Position Preferentially Adopts a Syn Base Conformation

To gain insight into the inefficient bypass across N7AcPhG by polη, we determined a crystal structure of polη in complex by templating N7AcPhG with a nonhydrolyzable dCMPNPP (dCTP*) in the presence of Mg^2+^ cofactor. The nonhydrolyzable nucleotide was used as it is isosteric to dCTP and its coordination to the active-site metal ions is essentially identical to that of dCTP [19]. The polη-N7AcPhG complex was crystallized in *P*6_1_ space group with cell dimensions of a = 98.785 Å, b = 98.785 Å, c = 81.876 Å, α = 90.00°, β = 90.00°, and γ = 120.00°. The structure was refined to 1.98 Å resolution with *R*_work_ and *R*_free_ values of 18.2% and 21.2%, respectively. The statistics for data collection and the refinement are summarized in Table 2.

**Figure 5:**
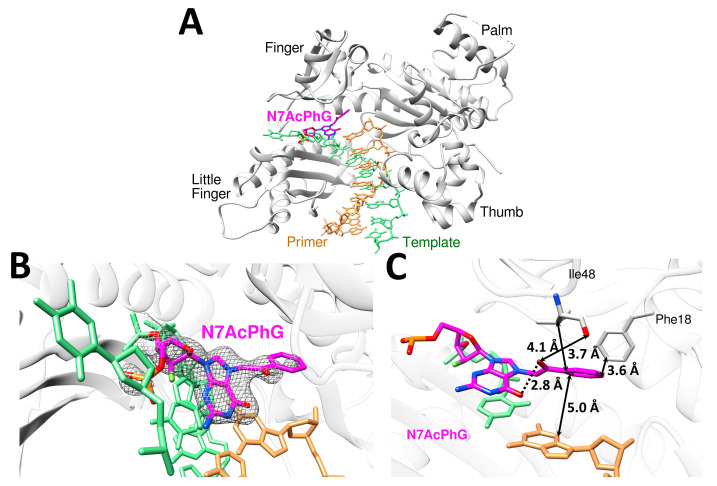
Structure of polη encountering *syn*-N7AcPhG at the templating position in the presence of a nonhydrolyzable dCTP analog and Mg^2+^ cofactor. (**A**) Overall structure of the polη-N7AcPhG DNA complex. Polη is shown in gray; template in green; primer in tan; and N7AcPhG in magenta. (**B**) Close-up view of the active site with the electron densities (2*F_o_*-*F_c_*) of *syn*-N7AcPhG contoured at 1σ. The electron density of incoming dCTP* is lacking, suggesting a transient binding of dCTP across N7AcPhG in the presence of Mg^2+^. (**C**) Stabilization of *syn*-N7AcPhG in the polη catalytic site. Distances between N7-acetophenone moiety and nearby bases and amino acid residues are indicated.

The overall structure of the polη-N7AcPhG complex displayed the four characteristic domains of Y-family polymerase [15]. The incoming dCTP*, however, was not observed in a catalytic site (Figure 5A), suggesting a transient binding of dCTP across N7AcPhG by the enzyme. The templating N7AcPhG adopted a *syn* conformation (Figure 5B) and acetophenyl moiety stretched to the binding site of the incoming nucleotide, thereby forming van der Waals contacts to Phe18 and Ile48 (Figure 5C). These contacts, which are rarely observed in the bypass of other DNA lesions by polη, could deter binding of dCTP at the replicating base pair site. Overall, the preferential adoption of *syn*-N7AcPhG conformers and unusual hydrophobic interactions in the polη catalytic site would greatly slow the incorporation of dCTP (Figure 5B). Despite numerous crystallization trials, it was not successful to obtain ternary complex crystals containing incoming dCTP*. Furthermore, substituting Mg^2+^ for Mn^2+^, which previously yielded polη-N7BnG:dCTP* ternary complex crystals [17], failed to produce ternary complex crystals of polη-N7AcPhG:dCTP*.

**Figure 6:**
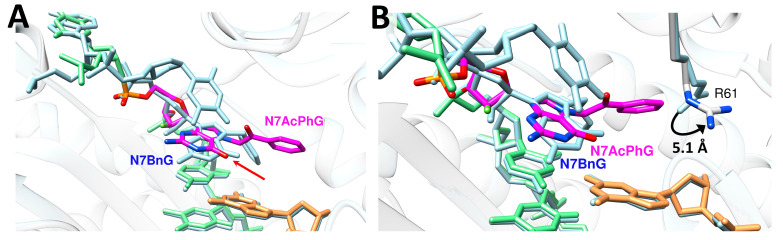
Comparison of the polη:*syn*-N7AcPhG and polη:*syn*-N7BnG structures. (**A**) Superposition of the polη-N7AcPhG (multi-color) and polη-*syn*-N7BnG (PDB ID: 7L69, light blue) [17] structures. The red arrow is pointing to the guanine O6 moiety of N7AcPhG and N7BnG. (**B**) Conformational differences of Arg61 in the N7AcPhG and N7BnG structures are shown. The guanidinium moiety of Arg61 in the N7AcPhG structure is pushed away, presumably to avoid steric clash with the acetophenone moiety.

We have previously reported that N7-benzylguanine (N7BnG) adopts a *syn* conformation in the replicating base pair site of polη in the presence of Mg^2+^ (Figure 6) [17]. The overall structures of the N7BnG and N7AcPhG complexes are very similar with the root mean square deviation (RMSD) of 0.159 Å over 427 α-carbons (C_α_). The conformations of the two templating lesions, however, were significantly different (Figure 6A). The phenyl moiety of N7BnG lies deep inside the pocket of the polη catalytic site, while the acetophenone moiety of N7AcPhG is projected toward the binding site of the incoming nucleotide. Compared to N7BnG, N7AcPhG is well-positioned to block the binding of an incoming dCTP (Figure 6A). This stretch of the acetophenone moiety toward the nucleotide binding site would cause the movement of the catalytically important Arg61 toward the major groove (Figure 6B). This conformational reorganization could deter the formation of ternary complex crystals of polη, N7AcPhG DNA, and dCTP even in the presence of Mn^2+^.

**Figure 7:**
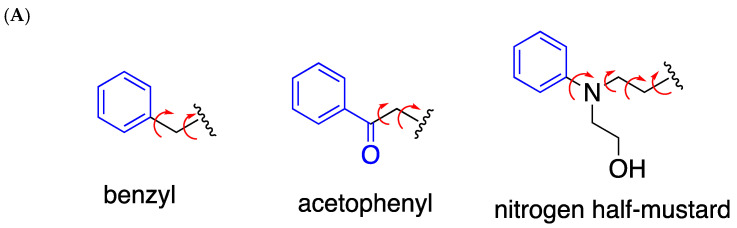

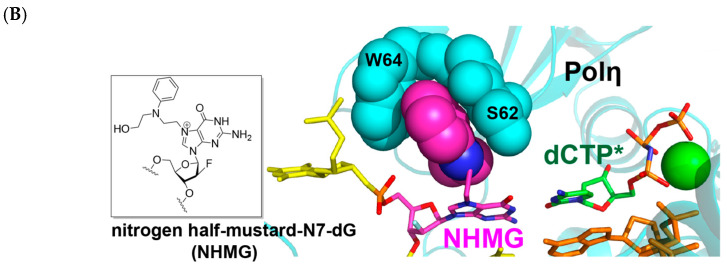
Conformations of N7-alkylG lesions. (**A**) Chemical structures of covalent adducts in N7BnG, N7AcPhG, and N7NHMG. π systems are indicated in blue. Rotatable bonds are shown as curved arrows. Note that the nitrogen half-mustard moiety is longer than the acetophenyl and benzyl moieties. (**B**) The conformation of NHMG in the polη catalytic site. The nitrogen half-mustard moiety of NHMG and the R61-W64 loop of polη are shown as spheres to highlight their interactions.

The conformational flexibility and stacking capability of N7-alkylG may influence the lesion’s base pair conformation at the templating position (Figure 7A). The previous observation that N7BnG can adopt both *syn*- and *anti*-conformations in the active site of polη, depending on the metal cofactor [17], could be explained by the relatively lower stacking capability of *syn*-N7BnG.

Despite extensive efforts, the *anti*-N7AcPhG conformation was not observed in the crystal structures with Mg^2+^, and crystals failed to form in the presence of Mn^2+^. This suggests that *syn*-N7AcPhG is favored over *anti*-N7AcPhG at the replicating base pair site, making the catalytically favorable anti-conformation less accessible during the bypass of N7AcPhG. This would lead to greater *K_m_* (10.2 μM (N7BnG) vs. 83.8 μM (N7AcPhG)) and lower *k_cat_* (56.4 × 10^−3^ s^−1^ (N7BnG) vs. 5.7 × 10^−3^ s^−1^ (N7AcPhG)) compared to N7BnG (Table 1). Similar results are observed in the case of the incorrect insertion of dTTP across N7AcPhG, displaying greater *K_m_* (51.7 μM (N7BnG) vs. 551.0 μM (N7AcPhG)) and lower *k_cat_* (20.6 × 10^−3^ s^−1^ (N7BnG) vs. 3.6 × 10^−3^ s^−1^ (N7AcPhG)).

The comparison of the polη-N7AcPhG structure with our reported structure of polη-nitrogen half-mustard-dG (NHMG) [18] provides insight into how polη uses varying strategies to bypass a wide range of N7alkylG lesions (Figure 7B). While N7AcPhG preferentially adopts a *syn* conformation at the templating position, NHMG is in an *anti* conformation, where the nitrogen half-mustard moiety engages in van der Walls interactions with the R61-W64 loop. The substantial difference in the *k_cat_* values (40.9 × 10^−3^ s^−1^ (NHMG) vs. 5.7 × 10^−3^ s^−1^ (N7AcPhG); Table 1) for correct insertion could be attributed to the relatively facile accommodation of NHMG by the R61-W64 loop [18], and a flexibility that might not extend to N7AcPhG. Note that nitrogen half-mustard moiety is longer and has greater rotational freedom than acetophenyl moiety (Figure 7A).

In summary, our kinetic and structural analyses, alongside reported mutagenicity studies of 2-chloroacetophenone, provide new insights into the effect of conformational flexibility and intercalation capacity of N7-alkylG lesions on their mutagenic potential. Unlike smaller lesions like N7-methylguanine, N7AcPhG tends to adopt a *syn* conformation in the active site of polη. This conformation, coupled with reduced rotational freedom due to coplanarity between the phenyl and carbonyl groups, leads to poor accommodation in the replicative base pair site of the polymerase. Although 2-chloroacetophenone can readily react with guanine to form bulky N7-alkylG adducts, these lesions would exhibit low mutagenicity as they are ineffectively bypassed by error-prone translesion synthesis DNA polymerases, aligning with the reported nonmutagenicity of 2-chloroacetophenone. The efficient removal of N7AcPhG by nucleotide excision repair, which excels at processing DNA-distorting lesions, would further mitigate its mutagenic effects. Our studies suggest that certain intercalatable N7-AlkylG lesions favoring *syn* conformation are likely to be less mutagenic due to their ability to deter nucleotide incorporation opposite the lesions.

## 3. Materials and Methods

**Synthesis of 2′-fluoro-2′-deoxy-N7AcPhG.** In a dry dimethylformamide (8 mL) solution of N2-phenoxyacetyl (Pac)-2′-fluoro-2′-deoxyguanosine (150 mg, 0.35 mmol) under argon atmosphere, 2-bromoacetophenone (1.4 g, 7.14 mmol) was added slowly until completely dissolved. The reaction mixture was then stirred at room temperature (25 °C) overnight (18 h) under argon atmosphere. Upon completion of the reaction, as monitored by thin-layer chromatography (TLC), the mixture was poured into 100 mL pre-chilled diethyl ether (Et_2_O), resulting in the formation of precipitates which were collected by filtration. The precipitate was subsequently dissolved in MeOH and concentrated to yield a white solid reaction mixture. This mixture was then subjected to silica gel column chromatography using a gradient of 0–12% MeOH in CH_2_Cl_2_ containing 0.5% triethylamine (Et_3_N). This purification process afforded 110 mg (60%) of 2′-fluoro-2′-deoxy-N7AcPhG. The resulting compound was analyzed using nuclear magnetic resonance (NMR) spectroscopy. 1H NMR and 13C NMR spectra were recorded on a Varian Mercury 400 (400 MHz) instrument. Peak multiplicities in 1H NMR spectra were abbreviated as s (singlet), d (doublet), t (triplet), and m (multiplet). 1H NMR (400 MHz, DMSO-d6) δ 9.96 (s, 1H), 9.25 (s, 1H, H-8), 8.08 (d, J = 7.5 Hz, 2H), 7.76 (t, J = 7.4 Hz, 1H), 7.63 (t, J = 7.6 Hz, 2H, aromatic H), 7.27 (t, J = 7.9 Hz, 2H, aromatic H), 6.89 (t, J = 8.5 Hz, 3H, aromatic H), 6.51 (dd, J = 15.4, 3.8 Hz, 1H, H-1′), 6.23 (s, 2H), 6.11 (d, J = 6.0 Hz, 1H), 5.34 (dt, J = 51.6, 3.4 Hz, 1H, H-2′), 5.11 (s, 1H), 5.03 (s, 2H), 4.45 (d, J = 17.4 Hz, 1H), 4.01–3.93 (m, 1H, H-4′), 3.68 (s, 1H), 3.63 (d, J = 4.4 Hz, 2H). 13C NMR (101 MHz, DMSO-d6) δ 191.38, 158.49, 151.06, 148.11, 136.34, 135.00, 134.10, 129.96, 129.87, 129.61, 128.64, 121.64, 121.13, 114.89, 114.87, 96.02, 94.10, 85.87, 83.96, 83.80, 73.31, 73.08, 67.99, 64.89, 60.93, 54.65, 52.24, 46.11.

**Protein expression, crystallization, and structure determination.** Polη was expressed and purified as previously described with minor modifications [20,21]. Briefly, polη was overexpressed in *E. coli* BL21(DE3) cells, cultures in LB media were grown at 37 °C until reaching the OD_600_ of around 0.7, and the cells were induced by supplying 0.3 mM isopropyl β-D-α-thiogalactopyranoside (IPTG). After further growing for 18 h at 20 °C, the pelleted cells (5000× *g* for 30 min) were resuspended in Ni–NTA column binding buffer A (50 mM sodium phosphate, pH 7.5, 500 mM NaCl and 10% glycerol) supplemented with 1 mg/mL lysozyme, 0.25% NP-40, 0.25% Triton X-100, and 0.25 mM phenylmethylsulfonyl fluoride (PMSF). After the resuspended cells were sonicated for 90 s, the lysate was centrifuged at 15,000× *g* at 4 °C for 40 min. The supernatant was then filtered through a 0.22 μm filter and purified through Ni–NTA column (GE Healthcare, Chicago, IL, USA). The elution fractions were pooled and further purified using the Heparin HiTrap column (GE Healthcare) followed by Superdex-75 size exclusion chromatography (GE Healthcare). The purity of the final product was confirmed by SDS-PAGE gel, concentrated, and the purified protein was flash-frozen in liquid nitrogen and stored at −80 °C for future use. To obtain the binary complex of polη-DNA complex, the N7AcPhG-containing 12-mer DNA was synthesized by Midland Certified Reagent Co. (Midland, TX, USA), and the primer (5′-AGCGTCAT-3′) was synthesized by Integrated DNA Technologies (Coralville, IA, USA). Polη was incubated with DNA substrate containing a 12-mer template (5′-CAT[N7AcPhG]CTCACACT-3′) and its complementary 8-mer primer (5′-AGTGTGAG-3′). Subsequently, a 10-fold molar excess of nonhydrolyzable dCMPNPP (Jena Bioscience, Jena, Germany, dCTP* hereafter) was added to the binary complex. Ternary polη-DNA complex co-crystals with nonhydrolyzable dCTP analogs paired with templating N7AcPhG were grown in a buffer solution containing 100 mM MES pH 6.5, 14–23% PEG2000 MME, and 5 mM magnesium chloride. Crystals were cryoprotected in mother liquor supplemented with 20% glycerol and were flash-frozen in liquid nitrogen. Diffraction data were collected at 100 K at the beamline 23-ID-D at the Advanced Photon Source, Argonne National Laboratory. All diffraction data were processed using HKL 2000 (https://www.hkl-xray.com/how-reference-hklhkl-2000, accessed on 15 May 2022) [22], and the structure was solved by molecular replacement using Molrep (Version 11.0/22/07.2010) [23] with polη structure with an undamaged DNA (PDB ID 4O3N) as a search model. The model was built using COOT (GTK4: 1.1.10) [24] and refined using PHENIX (version 1.20) [25]. MolProbity (http://molprobity.biochem.duke.edu/, accessed on 15 May 2022) was used to make Ramachandran plots [26]. All the crystallographic figures were generated using Chimera (version 1.11) [27].

**Steady-state kinetics of single nucleotide incorporation opposite templating N7AcPhG by polη.** Steady-state kinetic parameters for insertion opposite N7AcPhG by polη were determined as described previously [28]. Briefly, the oligonucleotides for kinetic assays (primer, 5′-FAM/GGGGGCTCGTAAGG-ATTC-3′ and template, 5′-CCGACT[N7AcPhG]GAATCCTTACGACCCCC-3′) were synthesized by Integrated DNA Technologies (Coralville, IA, USA) and Midland Certified Reagent company (Midland, TX), respectively. To prepare DNA substrate containing N7AcPhG, each oligonucleotide was annealed in a hybridization buffer (10 mM Tris-HCl pH 7.5, 1 mM EDTA). Enzyme activities were assessed using the reaction mixture containing 40 mM Tris-HCl pH 7.5, 60 mM KCl, 10 mM dithiothreitol, 250 μg/mL bovine serum albumin, 2.5% glycerol, 5 mM MgCl_2_, 80 nM primer/template DNA, and varying concentrations of incoming dNTP. To prevent end-product inhibition and substrate depletion from interfering with accurate velocity measurement, the enzyme concentrations and reaction-time intervals were adjusted for every experiment (less than 20% insertion product formed). The reactions were initiated by the addition of the nucleotides and stopped with a gel-loading buffer (95% formamide with 20 mM EDTA, 45 mM Tris-borate, 0.1% bromophenol blue, 0.1% xylene cyanol). The quenched samples were separated on 20% denaturing polyacrylamide gels. The gels were analyzed using Typhoon Phosphorimager (GE Healthcare) and ImageQuant software (version 8.1, GE Healthcare) to quantify product formation. The *k_cat_* and *K_m_* were determined by fitting the reaction rate over dNTP concentrations to Michaelis–Menten equation. Each experiment was repeated three times to measure the average of the kinetic results. The efficiency of nucleotide insertion was calculated as *k_cat_*/*K_m_*. The relative frequency of dNTP incorporation opposite N7AcPhG was determined as *f* = (*k_cat_*/*K_m_*)_[dN:N7AcPh]/_(*k_cat_*/*K_m_*)_[dN:dG]_.

## Figures and Tables

**Table 1:** Kinetic parameters for nucleotide incorporation opposite templating N7alkylG by polη.

Template:dNTP	*K_m_* (μM)	*k_cat_* (10^−3^ s^−1^)	*k_cat_*/*K_m_* (10^−3^ s^−1^μM^−1^)	*f* ^a^
dG:dCTP	2.7 ± 0.1	120.6 ± 6.0	45.6	1
dG:dTTP	159.3 ± 2.7	74.8 ± 0.9	0.47	1.0 × 10^−2^
N7mG:dCTP [16]	4.3 ± 0.4	56.4 ± 2.7	13.2	0.29
N7mG:dTTP	52.5 ± 1.7	49.3 ± 0.1	0.94	2.1 × 10^−2^
N7BnG:dCTP [17]	10.2 ± 2.3	20.6 ± 3.6	2.07	4.5 × 10^−2^
N7BnG:dTTP	51.7 ± 5.3	11.5 ± 0.3	0.22	4.8 × 10^−3^
N7AcPhG:dCTP	83.8 ± 6.5	5.7 ± 0.3	0.068	1.5 × 10^−3^
N7AcPhG:dTTP	551.0 ± 9.7	3.6 ± 0.3	0.0065	1.4 × 10^−4^

^a^ Relative efficiency:(*k_cat_*/*K_m_*)_[dNTP:N7-alkylG]_/(*k_cat_*/*K_m_*)_[dCTP:dG]_.

**Table 2:** Data collection and refinement statistics.

PDB CODE	Polη: N7-AcPhG (7LCD)
Data Collection	
space group	*P*6_1_
Cell Constants	
a (Å)	98.785
b	98.785
c	81.876
α (°)	90.00
β	90.00
γ	120.00
resolution (Å) ^a^	50–1.98 (2.05–1.98)
R_merge_ ^b^ (%)	0.057 (0.427)
<I/σ>	24.2 (2.5)
CC1/2 (2.05–1.98)	0.755
completeness (%)	99.9 (100.0)
redundancy	6.1 (5.9)
Refinement	
R_work_ ^c^/R_free_ ^d^ (%)	18.2/21.2
unique reflections	31,450
Mean B Factor (Å^2^)	
protein	32.39
ligand	40.55
solvent	36.56
Ramachandran Plot	
most favored (%)	97.9
add. allowed (%)	1.4
RMSD	
bond lengths (Å)	0.008
bond angles (degree)	0.968

^a^ Values in parentheses are for the highest resolution shell. ^b^ *R*_merge_ = Σ|I − <I>|/ΣI where I is the integrated intensity of a given reflection. ^c^ *R*_work_ = Σ|F(obs)-F(calc)|/ΣF(obs). ^d^ *R*_free_ = Σ|F(obs) − F(calc)|/ΣF(obs), calculated using 5% of the data.

## Data Availability

The atomic coordinate of Polη-N7-acetophenonedG complex has been deposited in the Protein Data Bank with the accession codes of 7LCD (htpps://doi.org/10.2210/pdb7LCD/pdb).
